# Social, economic and environmental implications of the COVID-19 pandemic

**DOI:** 10.3389/fpsyg.2022.898396

**Published:** 2023-01-24

**Authors:** Imran Maqbool, Muhammad Riaz, Umar Iqbal Siddiqi, Jamil Ahmed Channa, Muhammad Shahid Shams

**Affiliations:** ^1^Business Department, University of International Business and Economics, Beijing, China; ^2^Business Department, University of Okara, Okara, Pakistan; ^3^Business Department, Shah Abdul Latif University, Khairpur, Pakistan; ^4^Business Department, Kardan University, Kabul, Afghanistan

**Keywords:** climate change, coronavirus, de-globalization, economic disruption, intergenerational environmental consequences

## Abstract

The COVID-19 pandemic led to global lockdowns that severely curtailed economic activity. In this study, we set out to examine the social, economic, and environmental ramifications of the COVID-19 pandemic. This is a rare project that will have far-reaching consequences for the field. There are five sets of issues: short-term effects on oil and economic and agricultural policies, including regulations and COP26; long-term implications of monetary and fiscal intervention and investment in green agreements on future generations; prospects for further de-globalization and its effect on climate change and nature; and intergenerational environmental consequences, including debt and polling.

## Introduction

In late 2019, Wuhan, the capital of the province of Hubei, was the first place COVID-19 erupted and spread to the entire world, causing widespread devastation. Many governments responded by imposing lock-downs of varying intensities and durations. There have been numerous geographical and even international curfews in the past; tales of globally scattered lock-downs following World War II (Helm, 2020) suggest that they may not be able to combat disease transmission more effectively. On 11 March 2020, the World Health Organization (WHO) declared COVID-19 a “global Pandemic” and requested a vigorous response from the international community. By 16 April 2020, there were an estimated 1.99 million confirmed cases of COVID-19. The regional distribution and evolution of COVID-19 impede global economic development and have significant repercussions on public health (Bai et al., 2020; Lai and Deng, 2020; Sohrabi et al., 2020; World Health Organization, 2020). This number is 6.3% lower than the IMF's January 2020 World Economic Outlook projections.

The COVID-19 pandemic has exacerbated the world's affairs in various ways. For example, the rapid transmission of the virus across the globe has increased global economic uncertainty, which, in turn, has triggered financial and stock market volatility (McKibbin and Fernando, 2020). The world markets have changed rapidly from one state to another, and it is reasonable to assume that everything else has remained stable. Industrial substantiated reductions in air, road, and rail traffic have significantly decreased. Therefore, it is interesting to study what happens when such an abrupt transition occurs globally and domestically (Helm, 2020). What are the transitory consequences, and are they all likely to persevere? In fact, how remarkable are the reductions in GDP worldwide and city and air pollution? This is, to a large degree, anecdotal evidence of the impact of less travel on the wider daily environment, moving from a diminished tourism industry to a less unsettling untamed life influence. What does this tell us about the connection between GDP and these sporadic decreases?

What are the longer-term financial effects, particularly the ecological outcomes of the monetary stunts and their reactions? What will the degree of money-related improvements and the economic arrangements be? Will these include additional aid for “green arrangements” and related expenditure on greening transport, vitality, and horticulture? Will the coronavirus legacy be a firm or more fragile definition of COP26, sequential energy costs, and energy outskirt modification, or a more influential emphasis on lower vitality costs? What would be the effect of globalization, a hallmark focus of the world economy over the past 30 years? Would fears over fastenings gracefully spread and contribute to a deceleration in globalization or even a decline? In what capacity will the impact of the total reductions in GDP and the structural reactions to globalization happen? Will a continued decline bring beneficial results for the earth? Can the coronavirus help restore specific projects, notably horticulture, and a more specialized focus on household food production to the detriment of land use and even more earth-like nature? Eventually, will there be shifts in the behavior of mentalities toward the earth? Would the experience of the lockdowns encourage a step toward a higher value of natural products and businesses, indicating an old conviction that the world full of materialistic people (Helm, 2020).

### The short-term effects

In economies afflicted by the disease, the short-term effects of the techniques developed to halt its spread have been considerable. The inconvenience of lockdowns stunned various operations, with substantial repercussions for the development and travel industries, advertising, exchange, and the organization division. Although a couple of sections did well as people put away some products and went online shopping, the overall effect was seriously adverse. Due to a lack of experimental information, they choose the essential GDP impacts too soon. Nevertheless, various early IMF measures and others point to a phenomenal decrease that will continue as long as the lockdowns last and perhaps well into 2021 (International Monetary Fund, 2020). The natural effects right now are a lot harder to measure. However, others are substantially more promptly quantifiable because of the decline in ozone-hurting items released and the improvement in air quality. Ceaseless advances in settlement and ground-based mapping developments empower non-stop perception of different kinds of tainting, noticeable ozone-hurting substance transmissions, and urban air quality. Early signs are that heresy is genuinely down. Aside from China, Japan, and India, most economies have seen a significant drop in how much coal-fired power stations are used, especially in the early months of the pandemic in China.

**Figure 1:**
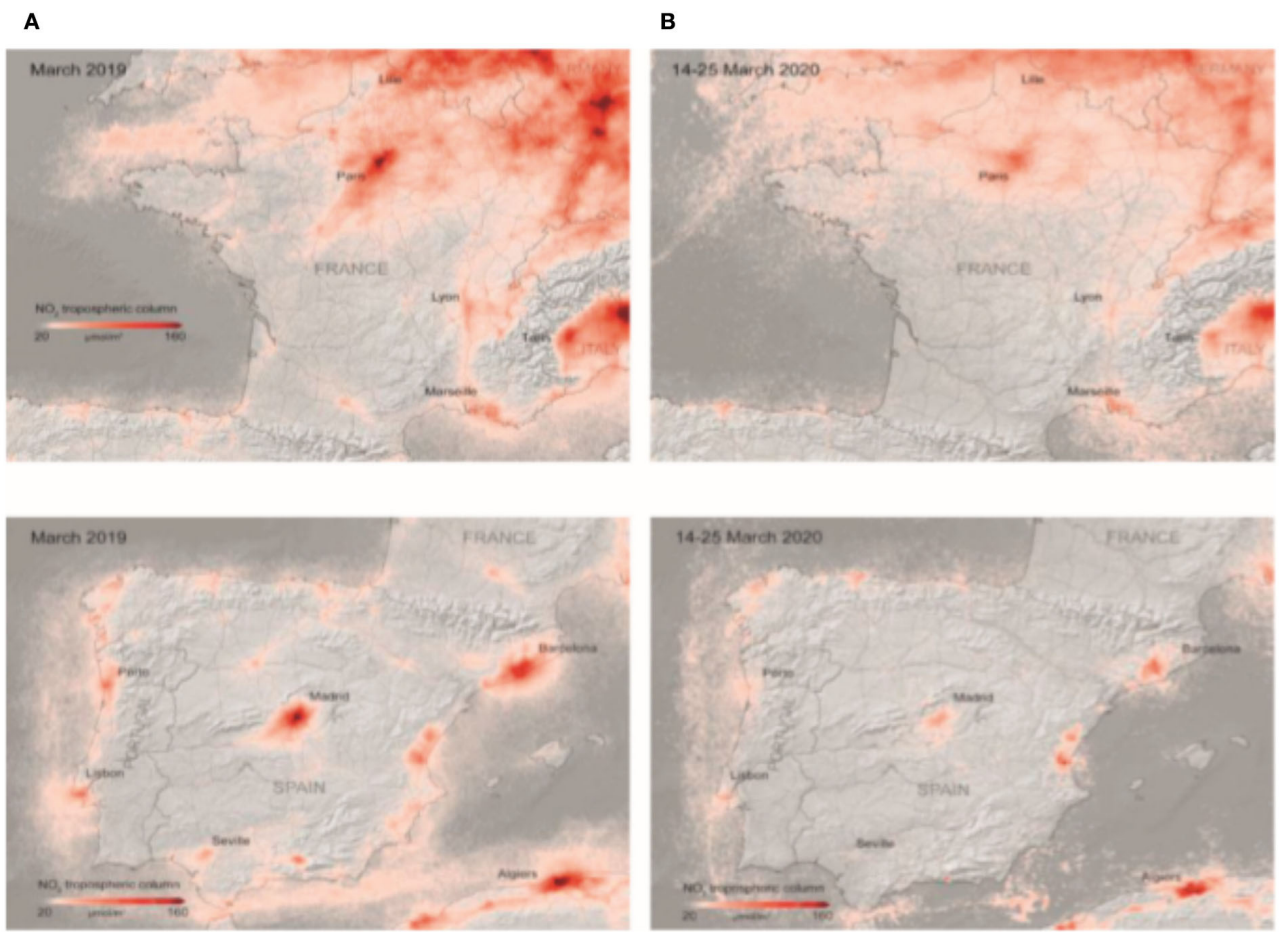
**(A, B)** Changes in NO_2_ emissions levels in France and Spain before March 19 and 14–25 March, 2020. Source: European Space Agency (2020).

**Figure 2:**
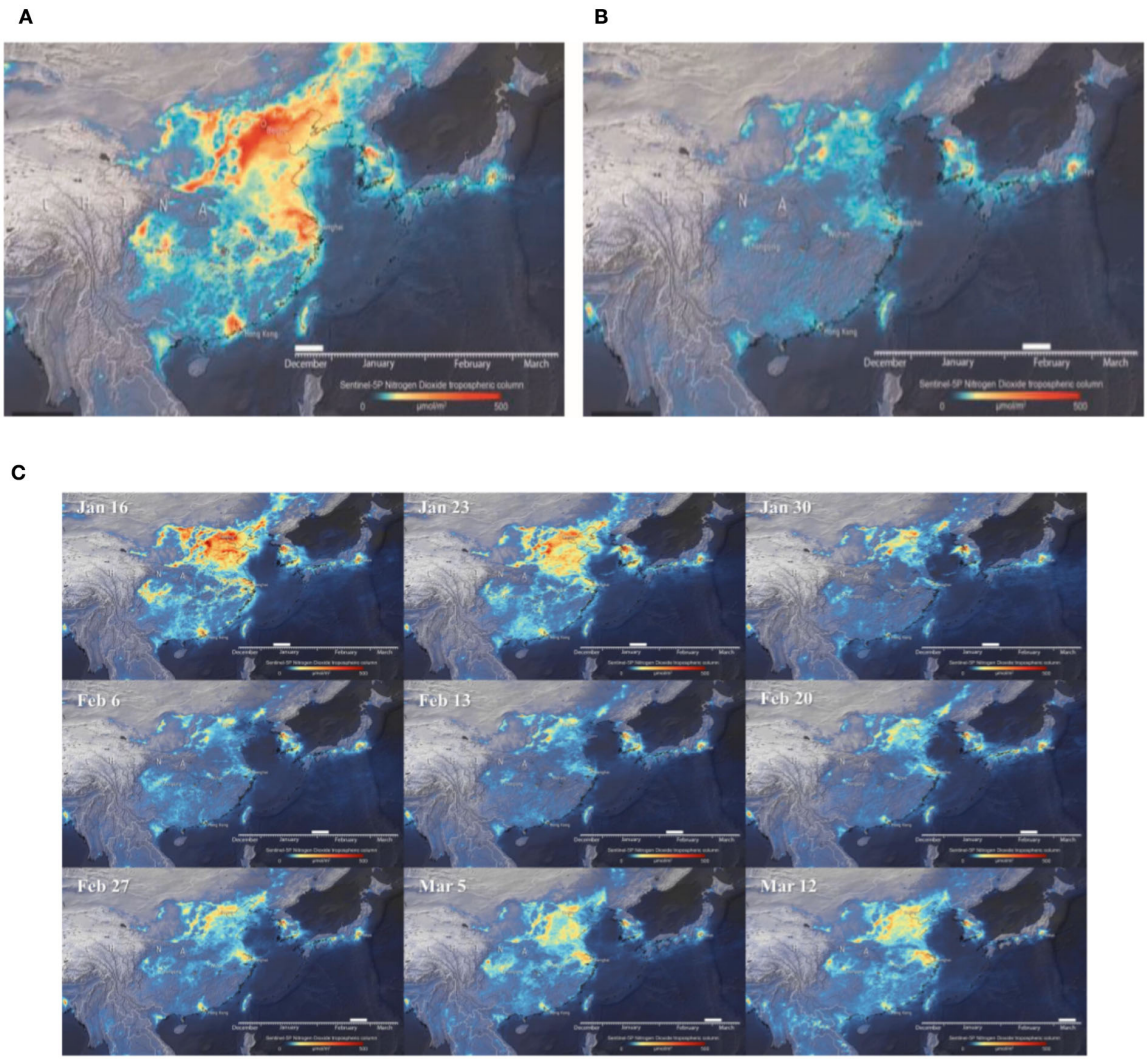
**(A–C)** Evolution of NO_2_ diminished levels in China. Source: ESA (2020).

**Figure 3:**
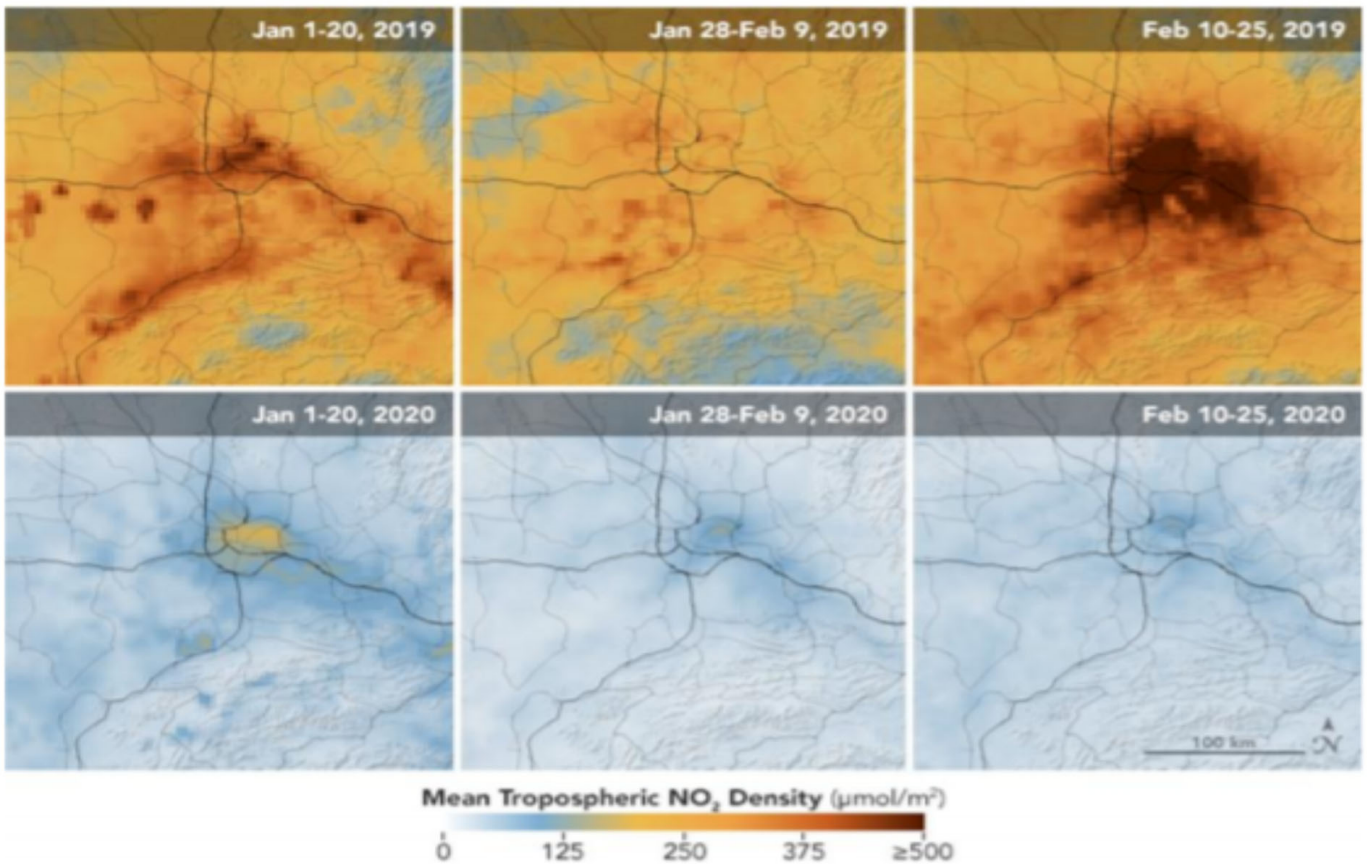
Changes in NO_2_ emissions in Wuhan, China. Source: NASA (2020).

There was an unexpected and sharp reduction in transport and oil consumption. However, these reductions in vitality-related discharges are not replicated in agrarian outflows, which by all accounts, do not appear to have been significantly affected so far. It is too early to set up exactly where the emanations have diminished. There are early signs, by and large, that nitrogen dioxide (NO_2_) discharges in numerous European industrial areas have dropped by almost half (European Environment Agency, 2020), overwhelmingly due to the breakdown of transport requests. In addition, a significant decrease in the levels of NO_2_ was also observed across France and Spain during March, 2019 and March, 2020 (Figure 1). Ozone-depleting material outflows dropped sharply in China in February, even with the beginning of a bounce back from late March. The scale of the drop may be ~20%. The reduction in pollution, caused by NO_2_, is reflected in Figures 2A, B. Similarly, the reduction in pollution, starting from Jan 16 to March 12, as shown in Figure 2C. Particularly in Wuhan, the NO_2_ emissions were reduced up to 30% during 2019 and 2020 (Figure 3). The most startling correlation in the pandemic was between considerable pollution reductions and a decline in aggregate productivity and marginal output.

Contrary to the statement that GDP and Emission are decoupled (IEA, 2016; European Commission, 2019), the COVID-19 pandemic influenced all the gigantic nations. China reported a 10 % decrease in GDP in the main long term of 2020 and a 6% rise (International Monetary Fund, 2020). Major economies in the EU may experience comparable or more prominent falls. The pandemic proves that decoupling has not happened worldwide or in Europe. Both contamination and GDP have declined fundamentally. The discharge to-GDP relationship is less significant than expanded climatic carbon fixation and GDP. Those focus numbers are not available for the main long periods of 2020. While emanations from power stations, modern concrete plants, and transportation are simpler to quantify, the centralization of carbon in the environment is the outcome of the net of all the various outflows (counting timberland consumption and corruption of peatland) soil and regular capital sequestration.

While the facts reveal that assessed nearby EU carbon discharges have been decoupled from EU GDP, it is evident that the EU yield and use structure has changed drastically. EU vitality yield decreases have been adjusted by EU carbon utilization in imports. It emphasizes the distressing reality that even the world's wealthiest nations cannot combat environmental change. The EU, in particular, has a static population while the rest of the globe continues to age rapidly. If global economic growth resumed its normal pace and populations worldwide began to increase, the situation would be much more challenging. Some parts of the territorial net-zero carbon discharge objectives in the UK and EU can be detrimental. In Europe, GDP dropped dramatically after the pandemic, proving that carbon cuts directly impact emissions and GDP. There is evidence from the correlation between emission reductions and GDP during pandemic lockdowns that suggests the world will not exceed the Paris Agreement's 1.5°C limit for abnormal climate change if GDP and population continue to grow at their current rates. This remark will trigger a conversation about whether the requested development will materialize. Predictably, the COVID-19 pandemic teaches us that the ecological outcomes of admission remain a crucial perspective in light of the need to slow the rate at which the global climate is changing, ensure clean air for everyone, and reduce the negative natural effects of increased consumption.

Biodiversity and ecological preservation comprise the second category of immediate impacts, for which the evidence is mainly anecdotal and based on foreseen consequences as opposed to a new study. Declining street traffic would decrease street execution and allow other species to flourish. Even though it may be difficult, this would improve natural security and regulations adherence. It will take less time to identify incidences of poaching, environmental violence, and contamination cases. As the northern hemisphere's breeding season for birds and mammals progresses, human protection for various species has decreased. Although domestic emissions are relatively simple (except those from wood, trees, and peat), the carbon content of fuels imported for consumption is a far more complicated story. For instance, UK carbon consumption is estimated to be 70% higher than carbon production (Environment, Food and Rural Department; Helm, 2017). The Committee on Climate Change (CCC-2019) stated that “by reducing the emissions generated in the United Kingdom to zero, we limit our contribution to rising global temperatures. We will never get to zero emissions (nor will we) because net zero does not stop the threat of climate change as long as there is pollution. Significant reductions in tourism will minimize footfall in vulnerable ecological areas and increase breeding success. Reducing roadside verge-cutting can support some plants, including native wildflower species. Natural resources will see rapid reductions in demand. For several reasons, lockdowns are less common in tropical areas, especially rainforests. Short-term ecotourism cuts would have the opposite effects, reducing emissions and increasing incomes that some countries depend on for protection, such as rainforests in Costa Rica and the Botswana Okavango Delta. On average, it is too early to determine the net consequences, and there is likely to be considerable variation between species, habitats, and countries. Given that emissions have fallen in the short term and are forecast to be slightly lower in 2020 as a result of the lockdowns, there is a temptation to argue that the need for immediate action is correspondingly less immediate and that other concerns, such as income security and welfare benefits, will take precedence over climate change in the short term and beyond.

Because of the current situation, strict environmental regulations have been relaxed. Due to lockdowns and social removal laws, the requirement has been dropped. The fuel quality is necessary for the US and consistency with the earth's confines (Utility Dive, 2020). In addition, when the lockup imperatives relaxed, falling oil and gas prices and decreased prerequisites created conditions for an psychological emotions turnaround in car demands and traffic-related emissions. If the explanation is that it costs investment funds for drivers now, it will have ramifications for prosperity in the end for both air quality and environmental change.

### The positive effect of COVID-19 on environmental quality

Air quality is fundamental for individuals' prosperity; however, 91% of the world's population currently resides in nations where it's at its worst (WHO, 2016). Environmentally-related deaths are at a tipping point due to pollution levels (Zhang et al., 2017). The WHO report for 2016 shows that this climate contamination is a leading cause of death around the globe. There has been a drastic reduction in available modes of transportation. Additionally, normal commercial activities have generally ceased.

### Long-term economic effects and some of their future environmental impacts

At first, the counterfactual should be used to assess the long-term consequences of the pandemic on GDP. This pollution raises an interesting question: what may have happened to GDP and development levels if the pandemic had not occurred? Although it is tempting to blame the pandemic for lower GDP levels and related products, by January 2020, a significant portion of the conditions for a financial exchange crash and downturn had only been established. It would be helpful if it were guaranteed that there was no budgetary emergency in the US, China, or EU, as the IMF (International Monetary Fund, 2020) asserts. This would be good because the positive effects on the markets from the absence of such an emergency have been greatly exaggerated. That is why fiscal arrangement activity was required to fight this mid-year of 2019. By January 2020, the EU was playing with a downturn, China's advancement rate was tumbling down, and indicators of global monetary growth were meager at best. In other words, a major economic downturn may have occurred regardless of whether or not the COVID-19 epidemic occurred. We cannot know whether that should have occurred. Another understanding of the counterfactual, with solid ecological ramifications, is recalling that oil prices fell before COVID-19 affected Europe or the United States, even before the extraordinarily diminishing popularity of oil.

Saudi Arabia's decision to increase requests precedes consideration of the pandemic's financial impact following the failure of discussions with Russia. Regardless, higher oil prices were anticipated to increase in 2020. There were valid reasons to expect oil prices to keep crashing throughout the following decade, as the flexible side was fortified by expanding global shale production. The exciting side is debilitated by decarburization and the approach of electric vehicles (Helm, 2017). Stocks cannot be reduced to a degree near a request to raise prices to 2019 levels despite actions by OPEC and OPEC+, such as the guarantee to reduce gradually by mid-April 2020. Demanding cheaper oil and gas will be met with a sluggish response, and future predictions from the oil showcase's perspective will warn capital products venture. The cheaper gasoline operating expenditures without a carbon balancing demand may increase the 2019 shift toward SUVs rather than electric cars. Quantitative tightening was also increased, with interest rates decreased and extra QE being implemented;

- External economic stimuli through expanded borrowing;
- Procurement strategy for ‘environment offers'-based programs.

### The monetary easing

Money-related facilitating began as a reaction to the dot-com emergency in 2000. Rather than pushing economies to withdraw after the long blast of the 1990's, national banks worldwide have chosen to push loan fees down to zero. Moreover, 20 years of monetary exceptionalism finished, with actual loan costs ostensibly and harmfully close to zero. This resulted in an escalating resource bubble in the housing market, followed by another stock market collapse in 2007–2008. Accordingly, money-related security was additionally reinforced with QE, increasing the asset reports of national banks. Negative monetary loan costs and QE helped resource costs and created another advantage bubble that, in the end, detonated during the 2020 pandemic. The more extensive macroeconomic effect of quantitative facilitation has been generally examined and regularly disputed. Its impacts have been less concentrated on the environment. When national banks purchase government and corporate securities, quantitative facilitating expands resource esteems, including properties, and fortifies the connection between present and future qualities by diminishing the time-rebate rate, prompting lower long government costs. Higher land costs change the financial aspects of planting and the temperature impact of farming. The higher the property price, the more prominent the allure of a little land that is inevitably brought into development instead of being left to nature. First, it suggests they have strengthened the CAP's negative atmospheric impact. The insignificant or zero absolute obligation load limits the hunger for sparing and the motivating force for spending. More use causes more waste and harms the environment. The related counterfactual is the thing that would have changed if actual financing costs surpassed the chronicled regular and approximated long-term development rate (Borio and Gambacorta, 2017).

If we assume the actual loan cost was about 2% between 2000 and 2020, the measure of obligation, investment funds, and salary would have differed, and the properties' valuation would have been much lower. If we suppose that money-related exceptionalism is saved or possibly exacerbated by the pandemic, we ought to hope to rehash everything that follows. Cost advantages will keep rising, obligation levels will increase, and investment funds will dominate utilization. Lower loan fees and QE will reduce venture capital expenses and significantly cut investment funds. The former will decrease the interest in sustainable power sources and the yield of atomic power, while the latter will be capital-intensive (as a rule, with zero immaterial expenses).

Even so, this expense of capital impact does not counterbalance the fall in vitality costs since it only applies to some innovations, not just low-carbon improvements. The effect would depend on what policymakers are doing, whether policymakers are changing the lower cost of spending financing into manageable and atomic vitality, and whether the significant oil expense is adjusted. What makes a difference is that ecological approaches regulate the money-related components and carbon taxes of national banks.

### Debt and fiscal stimuli

Monetary aid enabled every global economy to respond to the 2007/08 recession. In the end, obligation rates in China, the United States, and the European Union increased proportion to GDP. The quantitative easing program of the European Central Bank, which committed to “take the necessary actions” to reduce loan cost spreads, supported a decline in financing costs for all EU member states to historically low levels (Pisani-Ferry, 2014). However, in the second half of 2019, when the global financial outlook deteriorated, the majority of governments ceased deficit reduction efforts. The legal reason for the more prominent financial extension is regularly referred to as spending. It was argued that the venture, financed by the government, did not strengthen the hidden financial condition. As it were, a U-turn strategy has been broadly utilized. Even inside the EU or the United Kingdom, it has been asserted that the purpose of revising the general financial plan was politically wasteful and that the accounting report should be accorded a greater degree of importance, with a focus on spending, resource creation, and liabilities. (The United States has never attempted to restore its spending plan).

Fiscal measures in most EU nations, including Germany, have now fundamentally been expanded in the wake of the pandemic, and steps have been taken in the US and China. The EU has sold a EUR 500 billion pack (around USD 545 billion), whereas the US Federal Reserve reported a $2.3 trillion assortment. Any such cost would have been incurred as a result of the programmed stabilizers compensating for greater labor costs and lower charge assortments. These indicators are connected to rising total requests and have a Keynesian basis. From a financial perspective, the questions are about the impacts of GDP spending, the uniqueness in effects between utilization and speculation, how the segment of the expenditure is spent, and specifically, the ability of governments to manage environmental change and different endeavors to improve ordinary capital. What is essential to the economy is not the financial improvement (even though it impacts spending as examined above), but its arrangement and how it ponders the accounting report. Monetary gain will increase demand for power, transportation, and agricultural items. They will, in turn, expand their interest in wood and things from rainforests. There will be a quest for these meat items (from Amazon). Hardwood production (from the world's rainforests), hydroelectricity generation (from the watersheds of the world's major rivers), and palm oil production would all increase as a result (from Malaysia and Indonesia).

### Green tickets

Others argue that an open vehicle expenditure bundle is the key to being sought after with considerable interest and gently stunning the competition in light of the compositional influence of the money-related and financial improvements outlined above. A vital piece of this will be a cutting-edge environmental change pack, a green arrangement. Others called this another “Marshall Plan” green (European Commission, 2019, 2020). There are two plans: presumptions on the higher monetary gains of these activities, which are generally considered to have ecological moderation and distributional ramifications compared with different ventures, and ways to store and handle these costs. Interests in sustainable force, for instance, are typically characterized as either cost-serious compared to non-renewable energy sources or becoming so in the near future. This is a silly and risky proposition. If it is true, this venture will happen, at any rate, leaving no requirement for extra rewards and, subsequently, no need for a green offer. None of the patrons of the “Green Deals” will acknowledge the end.

On the other hand, if it is incorrect, the amusement for a green understanding essentially lies in the comparison between the more significant expenses of sun-powered vitality and the anticipated carbon value that would have satisfied the environmental change goals, particularly net zero. The least complex financial alternative to address this void would be the carbon cost, both at home and on the outskirts. Afterward, the green macroeconomic arrangement would again be pointless. What is missing is a purpose behind utilizing strategy endowments rather than improving the effectiveness of business costs on contamination.

If the inexhaustible were not on the path to solid cost intensity in 2019, the above-described dramatic declines in oil, gas, and coal prices would have altered the math further. The lobbyists for Greenpeace are quick to fight for acceptable force source expenses, but they are not eager to press the oil subsidiary dispute. Lobbyists for proficient power source energies will ordinarily fail to distinguish apples and oranges, ignoring atypical framework costs and restricted and disaggregated yield (Helm, 2017). The second point of contention applies to relative monetary returns. Expecting a lift to venture is the best reaction to the macroeconomic stuns between different kinds of speculation. The alleged focus on green investment has a long way to go. For instance, the arrival on street development can be as high as building houses and air terminals, especially if a carbon cost is calculated (Motorways for England, 2015; Highways England, 2019).

A hefty carbon tax will apply to vehicles powered by non-renewable energy sources, not hybrids. Despite security, availability, and fiber optics, the pandemic has revealed that the arrival of wellness consumption is considerably greater than predicted. They are the one part of the system whose economy has been improved by the pandemic. The switch to video and other remote availability and employment errands during lockdowns is enabled by the system's full-fiber favorable circumstances. It focuses on developing universal service obligation (USO), particularly in the UK and the rural US. Regardless of the duration, the government's total spending is minimal, necessitating trade-offs and decisions. The final consideration is where the project funding will come from, given that the tax reductions will likely encourage currency speculation. Green agreement proponents dispute if this will be future finance and inevitable investment funds or QE to adjust purchase prices. Some think about an enormous scope QE plan and then compare it with a Keynesian case by proposing that the resultant lift in total interest would be spread over the economy and pay for itself. This last section does not address every issue with quantitative easing (QE) while arranging usage overspending on foundation shortages to enhance the creation and impact of the universe of rising market demand and use by expanding carbon emissions and natural weights. Blending feasible ventures with Keynesian interest control is one of the most quarrelsome parts of green agreement circumstances. The case for the green hypothesis should be presented for an autonomous reason concerning a log jam, not as a lift to total interest. (This has excellent intergenerational impacts, which we will apply in section De-globalization, trade and economic outcomes below). In the post-lockdown era, there would be other compelling requests for national spending plans, regardless of the financial justifications for green planning as a part of a massive open consumption program.

## De-globalization, trade, and economic outcomes

In recent years, China's advancement and gigantic monetary development, estimated in regular terms of GDP, have directly and indirectly, exacerbated a major cause of ecological harm to the environment and biodiversity. China is the world's largest consumer of coal (IEA, 2019), damming the upper Mekong to provide hydropower. Each of the three principal streams has encountered monstrous defilement. Massive deforestation has emerged to flexibly hydropower, including all three fundamental streams. China's requirement for food and other characteristic assets has required substantial interest in Africa, empowering a massive scope foundation along its Belt and Road Network, manufacturing more dams and coal-terminated power plants, and opening common areas. Accordingly, considering the effects of the COVID-19 pandemic on China and its reactions is particularly significant. A few countries have attributed the majority of this contamination to assembling manufactured products, most notably by most EU nations and the US, representing a critical portion of the GDP on the planet. There has been a factual investigation of the Chinese development worldview, yet fares of carbon and vitality-concentrated products have been at their initial center (Pan et al., 2009). The end product of this was the relative decrease in the local creation of steel, compost, petrochemicals, aluminum, and even concrete (the significant five carbon-concentrated wares exchanged), somewhat displaced by Chinese fares in the US and especially in the EU. As it were, a considerable number of China's discharges have benefited customers in the US and the EU. Modest work permitted US and European firms to redistribute assembling to China and exchange the items to their home markets afterward. The US and the EU depend on everything from face veils and clinical instruments to interchange technology. According to some scientists, the COVID-19 pandemic will encourage a greater emphasis on residential development and consumer health, reducing contamination.

By 2019, remote communication progress will have slowed even further due to the introduction of new media, making it possible to examine the impact of the outbreak in a counterfactual manner for the first time. Robots are a supplementary workforce that does not tire, require payment for benefits, or contract the coronavirus. Monetary advancement will decouple from the idea of situating yield near modest information costs instead of customer proximity, with mechanical technology and 3D printing allowing jobs. They should take some pressure off the notions. Moreover, there is a general inquiry into the last connection between globalization and the impacts of the atmosphere. Second, the investigation is whether contact with this pandemic would cause more de-globalization. Second, there is the fascinating issue of whether a fortified house for security purposes is also good for nature. The two eco-explicit qualities of the general relationship are delivery and aeronautics-related natural outflows (counting foundation financing, port offices and ashore transport, additional traveler travel to deal with global supply chains, and extended travel industry because of China's globalized improvement). As has been noted, the famine has increased the apparent strength of country states over global establishments and their authority because of the hole in the factor input component (mostly coal and fert). Given that the United States has yet to elect a new president to quorate the World Trade Organization's appeals body, its operations have been suspended. The growing approval of state aid has also fueled protectionist campaigns. These impacts should be sufficient to raise the trade bounce-back, hence the shipping and aviation post-lock-down markets. Thus, the cumulative environmental effects of production at various locations must consider the other technological and ecological impacts. An additional positive impact of the more global trade strategy on the climate is that it should encourage a move toward reducing environmental costs and, in particular, a shift in border pollution. The European Union has already suggested a plan before the pandemic (European Commission, 2019).

Emissions and other environmental consequences are amplified by international trade, distorting the market. An even more skewed comparative advantage for China's imports of petroleum has been created by the low European energy costs (Helm et al., 2012). If China is willing to play by the rules, it is recommended that we return to the global carbon system. This was the scenario Obama found himself in. A major reason the US Senate did not ratify the Kyoto Protocol and has maintained an implicit veto over the signing of successive global warming treaties is the imbalance of power that emerged during the discussions. Agricultural lobbying groups, in particular, have used the outbreak as a springboard to push for stricter national supply controls and restrictions on international trade. The sustainability of supply chains is different from food self-sufficiency. The UK's lockdown experience has concentrated considerably more on food processing and logistics than food production. A carbon border tax decreases the manipulation of exports; the justification for expanded incentives to agricultural output is the retention of rentals and the protection of interests. The former improves economic outcomes; the latter does not.

## Economic facets of income between generations, an alternative to the balance sheet and natural resources

Concerning biodiversity, global change and misery are intergenerational problems. Given that it effects environmental change, it will gradually affect individuals. The current age may fare better with 1°C of warming due to the accumulation of assets in temperate places and the impact of modern warming on heating requirements, winter distress, and longer growing and harvesting seasons (Helm, 2015). Theft in biodiversity has continued to cause considerable financial misfortunes worldwide. We have an impressive inventory of biodiversity. For the time being, young people are projected to be disproportionately affected by the pandemic due to the rise in their use of travel services such as sports, movies, hotels, and travel agencies (Joyce and Xiaowei, 2020). Generally speaking, youngsters can avoid serious health effects from the pandemic itself, but the elderly (especially those over the age of 65) account for a disproportionately high number of deaths. In consequence, the youth will assume responsible fiscal behavior pertaining to the investment of resources. The response to the pandemic and a weakening of government resolve to deal with climate and biodiversity mischief are responsible for the production of such general pollution and obligation consequences, the different influence of responsibility and capital expansion on the coming century, and the likelihood of offsetting gains due to the addition of lower debt rates to projects as a result of financial improvements. With a steep discount rate, the future becomes increasingly important. It encourages long-term investment, but eventually, there will be a ceiling on open spending due to the depletion of wellbeing and social consideration consumption, and other buyers inviting initiatives to deal with the resulting financial crises.

For example, beneficiaries with higher approach expenditures and annuity payments are projected to be heavier in the present period due to profit and end-of-life care costs. A national accounting study represents the degree of intergenerational disparity and integrates usual properties. With emissions expenses assigned to those that generate discharges (the polluter-pay standard), ecological reforms ensure that signature capital expended out of 21 current spending records is preserved. Changes include contingency funds toward credit obligations. Those ventures (and not for actual use) would clarify the level of disparity. The scale of government spending and loans would likely lead to some accounting tricks, masking the figures. Although there would be constraints, taking spending off the balance sheet would not make them disappear. The traditional option was privatization, as the savings were associated with user fees. However, it is uncertain whether the government will increase its demand for electricity, water, transport, and even communications bills after the lockout to support these costs.

## The negative impact: Practical shifts

Reports have been released that interaction with coronavirus can affect behavior and personal and political decisions. Many hope that it will lead to more concerted COP steps and a greater ability to tackle biodiversity depletion. Others have the opposite view, arguing that lower sales would lead to access to short-term ventures and growth in business. Economic decision theory is the logical starting point for understanding how the consequences of the virus could affect behavior. One of the longest litigations in economic theory is the presumption of exogenous preferences and their transformation into a rational orientation ordered by the choice axioms. Exogenous assumptions and innovations form the basis of the neoclassical principle of demand and supply and the general equilibrium derivation. The clarification of why inferring exogenous impulses is critical is to separate psychology from economics. Once accepted, the specification only changes with adjustments to the data. It is pointless to change the underlying condition since it will go out. All of this has implications for environmental policies and COVID-19. That investigation has certainly become significantly easier. Increased visibility and awareness have contributed to a more widespread belief that lockdowns are occurring due to the pandemic and its coverage in the daily news and on social media (Shiller, 2019). The adage that “you don't know what you have until it's gone” applies: large numbers of people are now mindful of what the natural world means for us and what havoc its absence can wreak, as they are confined to their homes. These conflicting influences of education and new interactions may have far-reaching consequences. This is not the only effect the illness has had on us. As everyday professional competency assumptions were questioned and investment funds and profit projections were contested (Office of National Statistics, 2020), there was a great deal of disorder and optimistic uncertainty. Any food items quickly proved inaccessible and gradually disturbed the food chains. People started staving because of the resultant famine. Contradictory behaviors can dissuade individuals from exposing themselves to further dangers, while lower real and predicted profits will reduce the capacity to fund environmental changes. The investment base has shrunk, rendering the open fraction completely helpless and, for the most part, steadily rising fees. The challenge with examining is determining how much compensation is available for natural resources and initiatives (and saw instability). Due to the coronavirus, would reduce pay result in the intended improvement of ecological circumstances, or is it gradually determined by the likelihood that nature would be lost? More clearly, there are everyday properties, such as open-stop Gander. What are the highlights? The exogenous tilt hypothesis was met with notable snags, with clinicians emphasizing the likelihood of altering endogenous biases. For the data given, introducing a pandemic can make people pick sequential natural outcomes after some time, regardless of the new information. Have they developed a more prominent tenderness for nature? Given that the pandemic has affected everyone, will the experience make us bound to grasp political activity on various issues, including environmental change and COP, and a progressively lively network around us? Or do the reactions to the pandemic, on the other hand, encourage more patriotism, less globalization, and less society as people remain separated from social cooperation? Endogenous tastes face two challenges. First, human toxicity is a lengthy process in the best-case scenario. Human instinct has all the earmarks of being predetermined, and this is a reasonable assumption given the shape and purpose of the plan. Second, it is unclear whether changes in human behavior will prompt better desires for better natural outcomes. Is the reverse not the case? level of ecological discovery. We have numerous environmental issues, such as the creation of public resources and other open-weight targets for people who cannot withstand transition. It is unlikely now or in the future that an unnatural weather change of 3°C or more by 2,100 will be avoided by changing human behavior, regardless of whether it is conceivable or necessary. The reaction to the outbreak strongly indicates how important it is to focus on the facts rather than what others may like them to be. The cost of saving lives from this coronavirus should be measured relative to other premature deaths, including starvation, malnutrition, and diseases such as malaria. Prevention of such conflicts as those in Syria and Yemen is necessary. The pandemic, the basis of proactive environmental policies, demonstrates this partiality in human nature as an essential lesson. Such broader priorities, made possible by the pandemic, clearly resonate with concerns about climate change and the depletion of biodiversity. Stern and Taylor (2007) suggested that climate change economics would be measured based on zero potential discounting benefits. This critical presumption is necessary to conclude the economic viability of not taking action. Ramsey (1928) stated that because of the moment we live in, we do not differentiate against individuals. It is not conceivable: There is no evidence that human nature compels us to view these individuals in Yemen or Syria or those who perished from starvation in Darfur as comparable to our country's people. Aid allocations aim to reduce GDP by < 1%. It does not mean “finding” because if the “finding” does not reflect human action, it's an evil foundation for public policies on climate change or biodiversity. That will also slash through liberalism because liberalism is based on actual values, not idealized ones.

## Conclusions

The fight against coronavirus's aftereffects will go on for a long time. It could end up being one blip in the long struggle between individuals and infections in the history of humanity, even though individuals largely disregarded the Spanish Flu during the 1920's and 1930's. Individuals are worried about diseases and may need to determine how best to respond to this pandemic. While there have been immediate ecological gains from reductions in emissions and consequent improvements in air quality, a portion of these is likely to be short-lived as and when things normalize and GDP recovers, backed by significantly associated capital and financial boosts. Although it is essential not to incorporate its longer-term impacts, the coronavirus offers necessary proof for examining the causes of contamination and, in particular, the effects of a wide-ranging abrupt stop investigation on a significant amount of financial movement, especially on transport. The fact that the epidemic has not been uncoupled from pollution and GDP is its single most crucial fact. It is hard to overstate this activity's importance for devising a strategy.

We intended to mitigate environmental change and biodiversity loss. The rate of technological innovation over the next 30 years should be unsurpassed and accelerated by a fast-track initiative to convert capital stocks into global GDP growth of just 3 percent per year, another billion people, and a global temperature increase of only 1.5 degrees Celsius. To achieve mechanical success in critical biodiversity hotspots, especially the big rainforests, it will be necessary to account for and balance the typical degrading qualities. To create intergenerational inequality, macroeconomic strategies are likely to prioritize requests over investments, retain capital misestimation by quantitative easing (QE) and unfavorable actual loan payments, and promote wellbeing and associated transparent administration rather than conditions that will help the current generation—and especially the more experienced individuals—unnecessarily follow the needs of the future. It is difficult to discern the motivations behind assumptions. However, one stands out: the reduced assistance to globalization and the subsequent decreases in global carbon-scaled exchanges, as do the transport and avionics sectors and the related carbon-serious segments. For these potential points of interest, the pace of change from emerging developments that make driving and business-related travel targets less desirable should be applied, allowing for an even more carefully planned, competitive economy. While the total potential outcomes of the pandemic would not be ecologically sustainable, they do not deal with monetary disputes, environmental changes, and the unfortunate natural environment. The biological case aims to improve air quality, reduce marine emissions, and limit the loss of coal and peat, which is similar to the purpose behind transport zap. Coal and peat-eating misfortunes persist, as is the case with freight costs. This pandemic has essentially altered the points of interest, and bearing in mind that these alterations are insufficient to change our attitudes in the long run, they will help recalibrate the responses, and this is reinforced by the one significant advantage of coronavirus—an explosion of the logical realities of the realizations upon which we need to develop another approach to science.

## Author contributions

All authors listed have made a substantial, direct, and intellectual contribution to the work and approved it for publication.
